# Editorial: Immunotherapies against infectious diseases

**DOI:** 10.3389/fmed.2024.1426765

**Published:** 2024-05-16

**Authors:** Saba Tufail, Mohammad Asif Sherwani, Najmul Islam

**Affiliations:** ^1^Department of Genetics, University of Alabama at Birmingham, Birmingham, AL, United States; ^2^Department of Dermatology, University of Alabama at Birmingham, Birmingham, AL, United States; ^3^Department of Biochemistry, Jawaharlal Nehru Medical College (JNMC), Faculty of Medicine, Aligarh Muslim University, Aligarh, India

**Keywords:** immunotherapy, infectious diseases, viral diseases, COVID-19, emerging diseases, re-emerging diseases

Immunotherapy is employed to harness the body's natural defense mechanisms to treat/manage disease (1–3). The rise of emerging and re-emerging infectious diseases, coupled with the potential cancer risks associated with oncogenic infectious agents and the limited efficacy of existing treatments (4), has attracted attention of scientific community toward immunotherapy. Advancement in immunotherapies comprising vaccines, monoclonal antibodies, cytokines, T cells, and checkpoint inhibitors, holds significant potential in addressing not only cancer but infectious diseases as well. This Research Topic aimed to explore recent advancements in utilizing immunotherapy for the treatment and management of infectious diseases. We strived to incorporate studies that assess the efficacy of various immunotherapeutic approaches, including vaccines, in preventing, treating, and managing infectious diseases.

This Research Topic elaborates the current research on immunotherapies (including vaccines) against various infectious diseases. Among around 15 different types of submissions received, we were able to collect a total of 9 articles that includes 4 Original Research articles, 2 Brief Research reports, 1 Review article, 1 Minireview, and 1 Opinion article. These articles presented diverse infectious diseases, including emerging and re-emerging diseases. We were pleased to have original research articles exhibiting studies in cell culture, mice as well as patients. This indicates that the findings of this Research Topic range from pre-clinical to clinical studies and may likely benefit and be of interest to a wider scientific community. Moreover, the studies included in the Research Topic are focused on immunotherapies against various viruses, protozoa and bacteria. In the coming paragraphs, we briefly discuss the published studies in this Research Topic.

## Studies highlighting immunotherapies against viral diseases

Immunotherapeutic efforts to enhance antiviral immunity have been applied for quite some time (5–8), however, the present strategies need improvement to harness full potential of immunotherapy. Langan et al. present a novel immunotherapy strategy for against viral diseases, termed Artificial Immune Modulation Adoptive Cell Therapy (AIM ACT). This innovative approach utilizes AIM nanoparticles and is applicable to patients suffering from infections such as Epstein-Barr Virus (EBV), Human T-lymphotropic virus 1 (HTLV-1), human papillomavirus (HPV), or human immunodeficiency virus (HIV). In AIM ACT, the initial step involves collecting leukapheresis material from the patient. Subsequently, CD8^+^ T cells are enriched using paramagnetic AIM nanoparticles, which serve as artificial antigen-presenting cells (aAPCs) to amplify CD8^+^ T cells targeting key viral antigens. Following a 14-day expansion period, the AIM nanoparticles are removed, and the AIM ACT therapy is prepared for infusion into patients. Langan et al. present preclinical findings demonstrating the efficacy of this approach in expanding CD8^+^ T cells targeting viral antigens associated with Epstein-Barr Virus (EBV), Human T-lymphotropic virus 1 (HTLV-1), as well as high-risk Human Papillomavirus (HPV) types 16 and 18, utilizing healthy donor cells. The final AIM ACT formulations consists of a mixture of CD3+/CD4+ T cells, predominantly targeting various antigens from EBV, HTLV-1, or HPV, with over 90% of these T cells exhibiting the memory phenotype. Furthermore, EBV-specific AIM ACT cells exhibit functional activity, showcasing antigen-specific cytotoxicity and cytokine profile.

Immunocompromised individuals are highly vulnerable to opportunistic infections and malignancies. Traditional antiviral and antifungal medications often pose significant toxicity risks, exhibit limited efficacy, and can lead to long-term resistance development. The administration of pathogen-specific Cytotoxic T-Lymphocytes (CTLs) has demonstrated minimal toxicity and efficacy in combating infections such as Cytomegalovirus, Adenovirus, Epstein-Barr virus, BK Virus, and Aspergillus. However, this therapy faces significant limitations including regulatory challenges, high cost, and the absence of public cell banks. Conversely, CD45RA^−^ cells containing pathogen-specific memory T-cells offer a less complicated manufacturing and regulatory process, are more cost-effective, feasible, safe, and hold potential effectiveness. Sanz et al. introduce a therapeutic approach aimed at clearing viruses and particular fungi through donor lymphocyte infusions (DLIs) comprising CD45RA^−^ cells containing pathogen-specific memory T-cells from a healthy donor. The goal is to bolster and enhance the patient's cellular immunity until their own immune system is restored. They report preliminary findings from a study involving six immunocompromised patients; four afflicted with severe infectious diseases and two with EBV lymphoproliferative disease. Each patient underwent multiple infusions of safe familial CD45RA T-cells as adoptive passive cell therapy, comprising memory T-cells specific to Cytomegalovirus, Epstein-Barr virus, BK virus, and Aspergillus. Results indicate that utilizing familial CD45RA T-cells containing pathogen-specific cytotoxic T-lymphocytes represents a viable, safe, and potentially effective approach for treating severe pathogenic infections in immunocompromised patients via third-party donor sources.

Yu et al.'s report evaluates the status and pattern of antiviral therapy among outpatient cases of herpes zoster in China. They found that throughout the study duration, there was a progressive rise in the prescription of antiviral medications. Valaciclovir and famciclovir emerged as the primary prescribed antivirals, although their usage varied significantly across different hospitals. Conversely, prescriptions for acyclovir exhibited a declining trend. Despite not being recommended, the utilization of topical antivirals continued to increase. The annual expenditure remained consistent, attributed to the diminishing daily drug costs (DDCs) of valaciclovir and famciclovir. Overall, antiviral treatments closely align with contemporary recommendations, barring the utilization of topical antivirals. The insights gained from this study hold potential for optimizing healthcare resource allocation and herpes zoster management among Chinese outpatient populations.

The other interesting report by Godinez et al. details a descriptive analysis exploring user-generated social media dialogues on Reddit pertaining to FDA-approved HIV pre-exposure prophylaxis (PrEP) treatments. Examining thousands of Reddit posts, they identified 315 posts coded for PrEP, with 105 posts (33.33%) focusing specifically on user conversations regarding the transition of PrEP prevention. Notably, users displayed a keen interest in switching to emtricitabine and tenofovir alafenamide (Descovy), particularly citing concerns such as poorer adherence or existing side effects associated with emtricitabine and tenofovir disoproxil fumarate (Truvada). The analysis unveiled major themes including discussions on the cost disparity between Descovy and Truvada, apprehensions regarding side effects, changes in insurance coverage, and conversations surrounding the donation of Truvada to other users post-transition. These findings underscore the significance of leveraging social media platforms for digital pharmacovigilance, providing insights into emerging challenges related to HIV prevention, treatment, and adherence as experienced by patients.

In the opinion article by Alape-Girón et al., the authors highlight that preclinical data, alongside findings from completed clinical trials, warrant further exploration into the potential of equine pAbs as a broad-spectrum, cost-effective, and scalable treatment for COVID-19. They advocate for the comparability to antivenoms, suggesting that these therapeutics could be manufactured under Good Manufacturing Practices (GMPs) in low- and middle-income countries equipped with the necessary technology, and made available at prices feasible for economies with limited resources.

## Studies on immunotherapies against non-viral diseases

In addition to studies on anti-viral immunotherapies, this Research Topic also includes immunotherapeutic studies on human protozoa, bacterial and fungal pathogenic microbes. Phares et al. present findings on LD10, an active 18-amino acid derivative derived from a previously reported peptide (9, 10). *In vitro* experiments revealed that LD10 exhibited superior potency in disrupting PD-1 receptor signaling compared to LD01. Moreover, when administered prophylactically alongside an adenovirus-based malaria vaccine, LD10 treatment led to a greater expansion of antigen-specific CD8+ T cells secreting IFN-γ compared to LD01 treatment. Studies on dosing regimens established that a single dose of LD10 at the time of immunization with AdPyCS, a circumsporozoite (CS) protein of *Plasmodium yoelii*, was adequate to enhance the quantity of vaccine-induced antigen-specific T cells *in vivo*. Utilizing humanized mice with functional human CD8^+^ T cells, LD10-mediated modulation of human T cell responses was demonstrated. Furthermore, it was shown that LD10 could be expressed and secreted by a recombinant MVA vector, thereby enhancing the expansion of antigen-specific CD8^+^ T cells. These findings collectively establish LD10 as a potent immunomodulator that enhances T cell responses and supports the delivery of peptide-based immunomodulators via a viral vector.

Tahir et al. elaborate a survey-based study conducted across various countries to assess public acceptance of vaccines. They focused on the general population of Pakistan, aiming to evaluate their knowledge, attitudes, and practices regarding the Typhoid Conjugate Vaccine (TCV) and their willingness to receive the booster dose of TCV. Their findings revealed that while the Pakistani population possessed general awareness about the benefits of vaccination and the importance of booster doses, there was a lack of understanding regarding the availability and effectiveness of TCV provided by the government, particularly in combating typhoid fever. Despite a favorable inclination toward vaccination promotion, the study highlighted the persistence of certain religious and national misconceptions, underscoring the need for targeted intervention. However, the populace exhibited willingness to comply with booster doses of TCV, indicating an understanding of the importance of completing vaccine courses for disease prevention. The study suggests collaborative efforts among healthcare authorities, media outlets, and government officials to organize seminars and campaigns at both local and national levels, leveraging electronic and print media to address misconceptions about immunization, raise awareness about diseases, their consequences, modes of transmission, and the critical role vaccines play in disease prevention and saving lives.

Cubillos-Angulo et al. provide a comprehensive review examining the rationale and obstacles to the development and implementation of host-directed therapies (HDTs) against tuberculosis. Their analysis succinctly outlines medications that have completed or are currently undergoing evaluation in ongoing clinical trials. The study underscores the potential of combined multi-drug treatment with HDT in enhancing therapy effectiveness by reducing treatment duration, improving cure rates, and minimizing residual tissue damage. Additionally, Qadri et al. delve into recent advancements in immunotherapies targeting infectious diseases. They explore various immunotherapeutic approaches and their applicability in combating a wide range of infectious diseases caused by various bacterial and fungal pathogens.

In summary, the articles within this Research Topic gather insights from diverse experts in the fields of immunotherapy and infectious diseases, addressing various challenges and proposing potential solutions, thus enriching the field with new perspectives and understandings. The articles included studies from various countries of the world and on different pathogens, which further enriches our knowledge and opens avenues for further studies.

## Author contributions

ST: Writing – original draft, Writing – review & editing. MS: Writing – original draft, Writing – review & editing. NI: Writing – original draft.
